# The Overflow Effects of Movement Behaviour Change Interventions for Children and Adolescents: A Systematic Review and Meta-Analysis of Randomised Controlled Trials

**DOI:** 10.1007/s40279-024-02113-1

**Published:** 2024-09-18

**Authors:** Jie Feng, Wendy Yajun Huang, Chen Zheng, Jiao Jiao, Asaduzzaman Khan, Mehwish Nisar, Stephen Heung-Sang Wong

**Affiliations:** 1 Department of Sport, Physical Education and Health, Hong Kong Baptist University, Hong Kong, China; 2 Department of Sports Science and Physical Education, The Chinese University of Hong Kong, Hong Kong, China; 3 Dr. Stephen Hui Research Centre for Physical Recreation and Wellness, Hong Kong Baptist University, Hong Kong, China; 4 Department of Health and Physical Education, The Education University of Hong Kong, Hong Kong, China; 5 School of Health and Rehabilitation Sciences, The University of Queensland, Brisbane, QLD Australia

## Abstract

**Background:**

Considering the finite time within a 24-h day, the distribution of time spent on movement behaviours has been found to be associated with health outcomes.

**Objectives:**

This systematic review and meta-analysis aimed to summarise and evaluate the overflow effects of interventions targeting a single behaviour (physical activity, sedentary behaviour/screen time, or sleep) on other non-targeted behaviours among children and adolescents.

**Methods:**

Six databases (MEDLINE [Ovid], PsycINFO [ProQuest], EMBASE [Ovid], PubMed, Web of Science and SPORTDiscus [EBSCO]) were searched for relevant studies published before 13 May, 2024. Randomised controlled trials and clustered randomised controlled trials that targeted a single behaviour and also assessed the effects on non-targeted behaviours, comprised of healthy children under the age of 18 years, were included. Movement behaviours can be measured either objectively or subjectively. The revised Cochrane risk-of-bias tool for randomised trials was adopted to evaluate the risk of bias.

**Results:**

A total of 102 studies with 45,998 participants from 21 countries were identified, and 60 of them with 26,183 participants were incorporated into the meta-analysis. The meta-analysis demonstrated that physical activity interventions led to a reduction in the proportion of each day spent in sedentary behaviour (mean difference =  − 0.95% of wear time, 95% confidence interval − 1.44, − 0.45, *I*^2^ = 39%). Sedentary behaviour interventions resulted in increased standing time (mean difference = 3.87%, 95% confidence interval 1.99, 5.75, *I*^2^ = 0%). Interventions targeting screen time did not yield changes in physical activity or sleep. The findings on the effectiveness of sleep interventions on non-targeted behaviours and of physical activity interventions on sleep were inconclusive.

**Conclusions:**

Overall, the findings suggested that interventions aimed at increasing physical activity or reducing sedentary behaviour had overflow effects on non-targeted behaviours, but the effect sizes were small. Additional evidence is needed to reach definitive conclusions regarding the impact of behaviour change interventions on sleep and of the overflow effects of sleep interventions.

**Supplementary Information:**

The online version contains supplementary material available at 10.1007/s40279-024-02113-1.

## Key Points

**Table:** 

This systematic review represents the first attempt to synthesise evidence on the overflow effects of single movement behaviour interventions on non-targeted behaviours among children and adolescents.
An overflow effect on non-targeted behaviours was found in interventions targeting physical activity and sedentary behaviour.
Because of the risk of bias present in the included studies, cautious interpretation of the findings is needed.

## Introduction

The development of healthy movement behaviours (such as physical activity, sedentary behaviour, screen time, sleep) during childhood and adolescence has a strong association with a diverse array of health indicators [1–3]. Specifically, physical activity and sleep are positively associated with both physical (e.g. bone health, body weight status) and mental health among children and adolescents [4–6], and prolonged sedentary behaviour and screen time are associated with a higher risk of being overweight/obese and worse mental health among children and adolescents [7, 8]. Despite the importance of adopting healthy movement behaviours, previous systematic reviews have shown that children and adolescents worldwide engage in unhealthy lifestyles, characterised by low physical activity, high sedentary behaviour and/or screen time, and insufficient sleep [1, 3].

Given that the time in a 24-h day is finite and fixed, these component behaviours are co-dependent. The 24-h movement guidelines, which provide recommendations for the integration of physical activity, sedentary behaviour, and sleep within a 24-h period, have been proposed to recognise the interrelationships among these behaviours [9, 10]. The 24-Hour Activity Cycle, a holistic approach that treats all behaviours engaged in within a day as integrated, aligns with this framework [11]. Embedding the 24-Hour Activity Cycle in health promotion, previous studies using isotemporal substitution analyses have illustrated the relationships between time substitution and health outcomes. For instance, a meta-analysis covering 17,390 adults found that reallocating 30 min of sedentary behaviour to any intensity of physical activity was associated with lower levels of waist circumference, fasting insulin and the risk of all-cause mortality [12]. A systematic review of studies of children and adolescents revealed that substituting sedentary behaviour with moderate-to-vigorous intensity physical activity (MVPA) was associated with a lower risk of adiposity [13]. Furthermore, isotemporal substitution of physical activity, sedentary behaviour and sleep in a 24-h day has been found to be associated with improved physical fitness [14], motor skills [15], inflammatory markers [16], cognitive function [17] and mental health [18] in cross-sectional and longitudinal studies of children and adolescents.

Interventions targeting the improvement of all three behaviours across a 24-h day are still in their infancy, and most interventions are designed to change a single behaviour. The only intervention study that aimed to change adolescents’ physical activity, sedentary behaviour and sleep simultaneously adopted a quasi-experimental design in a school setting [19]. It was found that the intervention group had higher physical activity (both light-intensity physical activity and MVPA), lower sedentary behaviour and longer sleep duration after 1 academic year than the control group [19]. However, interventions designed to target a single behaviour have also been found to impact non-targeted behaviours. This phenomenon has been described as the overflow effect [20]. For instance, a systematic review summarising behavioural interventions for children under 5 years of age reported that a physical activity intervention had an overflow effect of decreasing sedentary behaviour [21]. Interventions aimed at reducing sedentary behaviour have reported improvement in physical activity (e.g. frequency of exercise and participation in sports, MVPA, steps per day) and sleep [22–24]. Regarding sleep, which constitutes a considerable portion of the 24-h cycle, the evidence for an overflow effect of sleep interventions is sparse and mixed. For example, an intervention targeting sleep routines among young children found an increased MVPA of 11 min per day in the intervention group compared with the control group, although the difference was not significant [25]; another family-based sleep intervention among infants and toddlers found no significant effect on light-to-vigorous physical activity [26]. Another intervention reported that adolescents spent less time in sedentary behaviour when they were instructed to increase sleep duration [27]. Given these mixed findings, it is imperative to conduct a review covering a wide age range (< 18 years) to systematically summarise the evidence of overflow effects in behavioural interventions. This review offers crucial evidence to better understand how behaviours interact in a 24-h day, which in turn can inform future interventions.

This systematic review examined the overflow effects of interventions targeting a single movement behaviour on other non-targeted movement behaviours in children and adolescents aged under 18 years. The secondary purpose was to investigate whether the overflow effects vary across different characteristics of the intervention (e.g. age group, setting, duration of intervention).

## Methods

### Protocol and Registration

The protocol of this review was registered in the International Prospective Register of Systematic Reviews (PROSPERO; CRD42022315153). This report follows the Preferred Reporting Items for Systematic Reviews and Meta-Analyses (PRISMA) guidelines [28].

### Information Sources and Search Strategy

A systematic search was conducted on 7 March, 2022 and updated on 13 May, 2024, using the databases MEDLINE (Ovid), PsycINFO (ProQuest), EMBASE (Ovid), PubMed, Web of Science and SPORTDiscus (EBSCO). Details of the search strategy are available in Table S1 of the Electronic Supplementary Material (ESM).

### Eligibility Criteria

The search had no limitations on publication date, but was limited to studies published in English in peer-reviewed journals. Only randomised controlled trials and clustered randomised controlled trials were included. Studies that included apparently healthy children aged under 18 years were eligible. Studies that targeted a single movement behaviour (physical activity of any intensity, sedentary behaviour, screen time or sleep) but also assessed the effects of the intervention on non-targeted behaviours were included in the review, for example studies that targeted physical activity but assessed the effects of the intervention on sedentary behaviour, screen time and/or sleep. Physical activity, sedentary behaviour and sleep can be evaluated using either device-based measures (e.g. accelerometers, pedometers) or subjective measures (e.g. questionnaires, interviews, diaries); screen time can be assessed through subjective measures such as questionnaires, interviews or diaries. Studies had to include a control group that did not receive any intervention or received an intervention that was not related to the content of the intervention group. For inclusion in the review, studies had to measure the outcomes of at least one non-targeted movement behaviour (physical activity, sedentary behaviour, screen time or sleep) at both baseline and post-intervention. Studies were excluded if they were (1) non-randomised controlled experimental studies; (2) qualitative studies; (3) case studies and case series; (4) grey literature; (5) comments/editorials; (6) reviews; or (7) studies targeting population with clinical diagnoses (with the exception of obesity/overweight).

### Study Selection and Data Extraction

After removing duplicates, two independent reviewers (JF, CZ) screened the titles and abstracts in the initial sample, followed by full-text screening of papers identified in the previous step. Any discrepancies were resolved by discussion or by consulting a third independent reviewer (WH). Data extraction (e.g. author, publication year, country, study design, population, setting, targeted behaviour, non-targeted behaviour, measurement of non-targeted behaviour, main findings, intervention duration) was conducted by two reviewers (JF, MN) and checked by a third reviewer (CZ). The targeted behaviour was determined by the purpose claimed by the authors and strategies of the intervention. The corresponding authors of potentially relevant studies were contacted to provide supplementary data. For example, for interventions targeting physical activity that used accelerometers, sedentary behaviour data expressed as minutes per day were requested, as it was expected that such data were collected; 52 e-mails were sent requesting such information, and 20 replies were received.

### Risk of Bias and Publication Bias

The revised Cochrane risk-of-bias tool for randomised trials (RoB 2) was used to assess the risk of bias in the included studies [29]. The assessment was conducted by JF and checked by JJ. This tool evaluated five domains, namely randomisation, deviations from intended interventions, missing outcome data, measurement and selection of reported results [29]. Discrepancies, if any, were resolved through consensus discussions or involvement of a third reviewer (WH) when necessary. Moreover, publication bias was visualised using funnel plots for meta-analyses that included a minimum of ten studies.

### Data Synthesis

Meta-analyses were performed for the post-intervention outcomes for non-targeted behaviours, using Review Manager version 5.4 [30]. Mean values and standard deviations were determined to estimate the effect of the intervention, and standard errors and 95% confidence intervals (CIs) were converted to standard deviations [31]. The pooled effect sizes were presented as the mean difference (MD; intervention group vs control group) with 95% CI. Random-effect models were performed, and a series of sensitivity analyses were conducted by removing studies one by one. Sub-group analyses were conducted based on various factors, including age groups (preschoolers, school-aged children, adolescents), settings, duration of intervention, effectiveness on the target behaviour (based on the statistical analysis results), whether the design of the intervention was based on a theoretical framework, wear time of accelerometers and risk of bias. Subgroup analyses were conducted if there were at least two studies examining the overflow effect on the same non-targeted behaviour within the subgroup. Heterogeneity was presented as *I*-square (*I*^2^) values and was categorised as low (< 25%), moderate (26–50%) or high (> 50%) [32].

## Results

### Study Selection

**Fig. 1:**
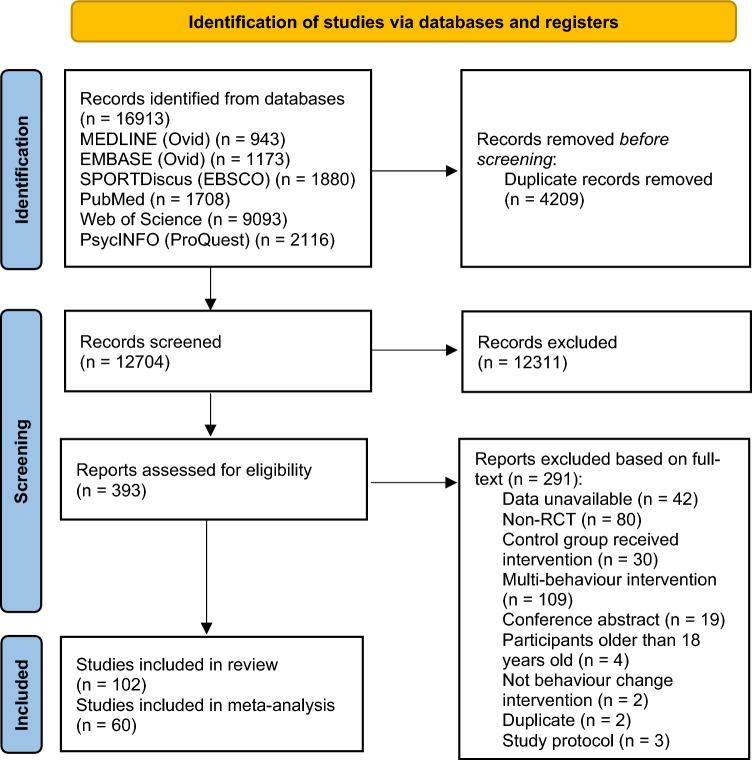
Preferred Reporting Items for Systematic Reviews and Meta-Analyses (PRISMA) flow diagram. *RCT* randomised controlled trial

After removing duplicates, 12,704 studies were included in the sample for title and abstract screening. Among these, 393 studies underwent full-text screening, 102 studies were included in the final sample, and 60 of them were included for the meta-analysis. A flow diagram of the selection process is presented in Fig. 1.

### Descriptive Characteristics of Included Studies

Table S2 of the ESM provides an overview of the studies in the final sample. A total of 102 studies covering 45,998 participants were included in this study. The average age of the participants in the studies ranged from 3 months to 17.9 years, representing a diverse group from 21 countries. Among the 87 physical activity (i.e. physical activity as the targeted behaviour) interventions, 68 measured sedentary behaviour, 16 measured screen time and seven measured sleep duration as non-targeted behaviours. All of the sedentary behaviour interventions (sedentary behaviour as the targeted behaviour; *n* = 6) measured physical activity as one of the outcomes, and eight interventions targeting screen time measured physical activity; three of the latter also measured sleep. Two sleep (targeted behaviour) interventions measured physical activity, one of which also reported sedentary behaviour.

### Overflow Effects of Physical Activity Interventions on Sedentary Behaviour/Screen Time and Sleep

The results of the 68 physical activity interventions that also measured the effects on sedentary behaviour [33–100] are summarised in Table S2 of the ESM. Nineteen studies reported significantly lower sedentary behaviour in the intervention groups than in the control groups at post-intervention [73–77, 79–88, 90, 91, 95, 96]; no significant difference was found between the intervention and control group in 46 studies [33–72, 78, 89, 97–100]. The remaining three studies solely reported within-group differences and did not examine the differences between groups [92–94].

**Fig. 2:**
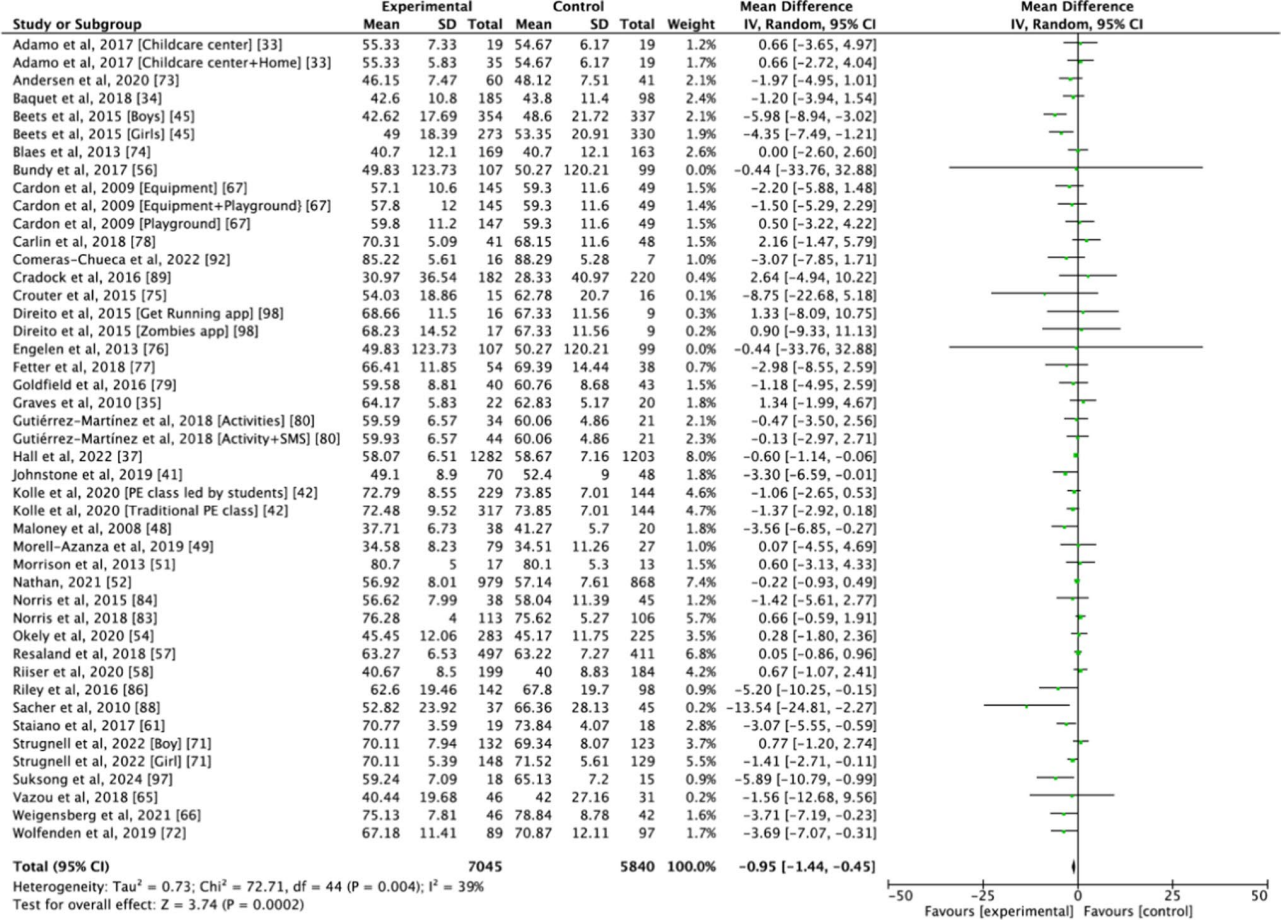
Pooled analysis on the effect of physical activity intervention on sedentary behaviour (% of wear time). *CI* confidence interval, *IV* inverse variance, *PE* physical education, *SD* standard deviation, *SMS* text messages

Thirty-seven studies examined the effect of physical activity interventions on sedentary behaviour measured as a percentage of wear time (Fig. 2) [33–35, 37, 41, 42, 45, 48, 49, 51, 52, 54, 56–58, 61, 65–67, 71–80, 83, 84, 86, 88, 89, 92, 97, 98]. All studies except one [88] used device-based measurement. The intervention groups reported less sedentary behaviour than the control groups (MD =  − 0.95%, 95% CI − 1.44, − 0.45, *I*^2^ = 39%). Subgroup analyses (Table 1) were performed based on participants’ age, setting, intervention duration, the effectiveness of the intervention on the target behaviour (physical activity), whether the study was theory based, wear time of accelerometers and level of risk of bias. The results indicated that physical activity interventions were effective in reducing sedentary behaviour among school-aged children and adolescents (school-aged children: MD =  − 0.88%, 95% CI − 1.53, − 0.24, *I*^2^ = 52%; adolescents: MD =  − 1.64%, 95% CI − 2.61, − 0.68, *I*^2^ = 0%), but were not effective among preschoolers (MD =  − 0.82%, 95% CI =  − 1.90, 0.26, *I*^2^ = 0%). Interventions conducted in schools and childcare centres observed significantly reduced sedentary behaviour (MD =  − 1.03%, 95% CI − 1.58, − 0.47, *I*^2^ = 44%). The pooled results indicated that the physical activity interventions led to a decrease in sedentary behaviour regardless of their effectiveness in improving the target behaviour. However, a larger effect was observed when the interventions effectively changed the target behaviour than those did not (effective: MD =  − 2.05%, 95% CI − 3.10, − 1.01, *I*^2^ = 49%; not effective: MD =  − 0.43%, 95% CI − 0.75, − 0.10, *I*^2^ = 0%). Regarding the use of a theoretical framework (theory based, no theory), intervention duration (less than 12 weeks, at least 12 weeks), wear time of accelerometers (whole day, segmented time) and the risk of bias (some concerns, high risk), significant decreases in sedentary behaviour were observed in all subgroups.

**Table 1:** Subgroup analysis on the effect of physical activity intervention on sedentary behaviour (% of wear time)^a^

Subgroup	Number of studies	Mean difference (% of wear time)	95% CI		*Z*	*P*-value	Heterogeneity within subgroups		
			Lower	Upper			*χ*^2^	*I*^2^ (%)	*P*-value
Child age									
Preschoolers	6	− 0.82	− 1.90	0.26	1.48	0.14	6.79	0	0.56
School-aged children	25	− 0.88	− 1.53	− 0.24	2.69	< 0.01	56.32	52	< 0.01
Adolescents	4	− 1.64	− 2.61	− 0.68	3.34	< 0.01	3.88	0	0.57
Setting									
School and childcare centre	27	− 1.03	− 1.58	− 0.47	3.64	< 0.01	55.60	44	< 0.01
Family	6	− 0.86	− 3.37	1.66	0.67	0.51	10.18	41	0.12
Community	3	− 0.43	− 2.41	1.55	0.42	0.67	5.32	44	0.15
Intervention duration, weeks									
< 12	13	− 1.15	− 2.20	0.09	2.12	0.03	20.89	23	0.18
≥ 12	22	− 1.06	− 1.73	− 0.40	3.13	< 0.01	49.99	50	< 0.01
Effectiveness on the target behaviour									
Effective	24	− 2.05	− 3.10	− 1.01	3.85	< 0.01	48.76	49	< 0.01
Not effective	13	− 0.43	− 0.75	− 0.10	2.58	0.01	16.63	0	0.55
Whether based on theory									
Theory based	19	− 1.06	− 1.84	− 0.27	2.65	< 0.01	51.77	59	< 0.01
No theory	18	− 0.69	− 1.18	− 0.20	2.75	< 0.01	20.85	0	0.53
Wear time of accelerometers									
Whole day	26	− 0.55	− 1.04	− 0.06	2.19	0.03	38.34	22	0.14
Segmented time	10	− 2.01	− 3.27	− 0.74	3.10	< 0.01	26.21	54	0.01
Risk of bias									
Some concerns	21	− 1.00	− 1.67	− 0.32	2.88	< 0.01	45.39	41	0.01
High risk of bias	16	− 0.94	− 1.73	− 0.14	2.31	0.02	27.04	41	0.04

*CI* confidence interval

^a^Based on 37 studies, of which 36 utilised device-based measures of sedentary behaviour

**Fig. 3:**
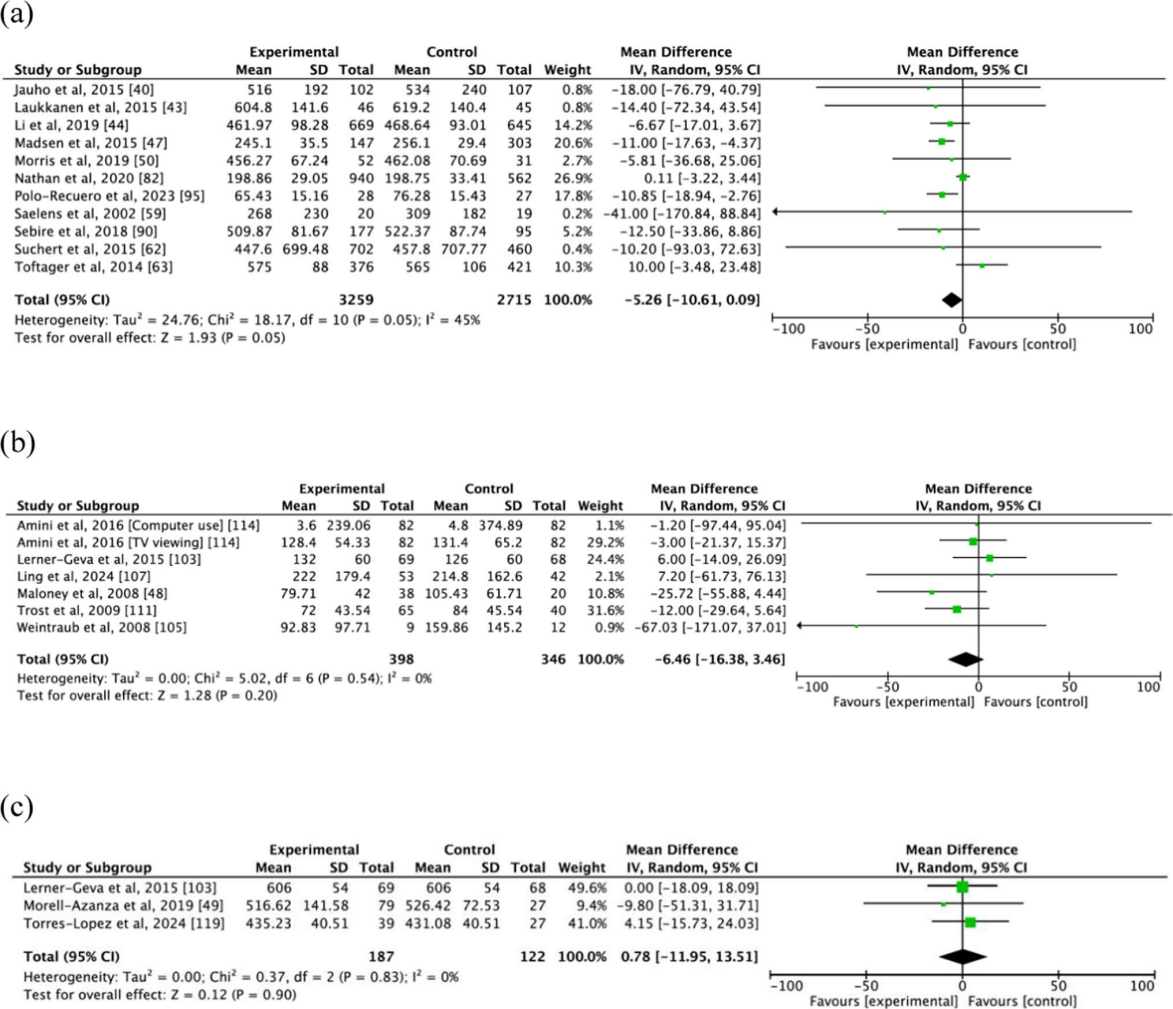
Pooled analysis on the effect of physical activity intervention on **a** sedentary behaviour (min/day), **b** screen time (min/day) and **c** sleep (min/day). *CI* confidence interval, *IV* inverse variance, *SD* standard deviation

Among the 68 physical activity interventions that also measured the effects on sedentary behaviour, 11 examined the pooled results of physical activity interventions on sedentary behaviour measured as minutes per day, and their meta-analyses results are presented in Fig. 3a [40, 43, 44, 47, 50, 59, 62, 63, 82, 90, 95]. There was no significant difference between the intervention and control groups (MD =  − 5.26 min, 95% CI − 10.61, 0.09, *I*^2^ = 45%), for both school-aged children (MD =  − 5.35 min, 95% CI − 12.19, 1.48, *I*^2^ = 58%) and adolescents (MD =  − 5.08 min, 95% CI =  − 16.23, 6.08, *I*^2^ = 34%). A sub-group analysis indicated that physical activity interventions were effective in reducing sedentary behaviour when measured subjectively (MD =  − 11.09 min, 95% CI − 19.05, − 3.13, *I*^2^ = 0%), but were not effective when adopting device-based measurement (MD =  − 3.84 min, 95% CI − 10.20, 2.53, *I*^2^ = 56%). Of the 16 studies that examined the effect of physical activity intervention on screen time [48, 101–115], nine reported decreased screen time in the intervention group [101, 106–113] compared with the control group, and seven did not find significant differences [48, 102–105, 114, 115]. Six studies met the meta-analysis criteria, and all of them utilised subjective measurement to assess screen time [48, 103, 105, 107, 111, 114] (Fig. 3b). No significant differences in screen time were found between the intervention and control groups in the meta-analysis (MD =  − 6.46 min, 95% CI =  − 16.38, 3.46, *I*^2^ = 0%).

Seven studies examined the effect of physical activity interventions on sleep duration [46, 49, 103, 116–118]; six of them did not find significant differences in sleep duration between the intervention and control groups [46, 49, 103, 116, 118, 119], while one reported significantly longer sleep duration in the intervention group than in the control group [117]. As shown in Fig. 3c, the meta-analysis showed no significant difference between the intervention and control groups (MD = 0.78 min, 95% CI − 11.95, 13.51, *I*^2^ = 0%), based on three studies that used both device-based [49, 119] and subjective measures [103].

### Overflow Effects of Sedentary Behaviour Interventions on Physical Activity and Sleep

**Fig. 4:**
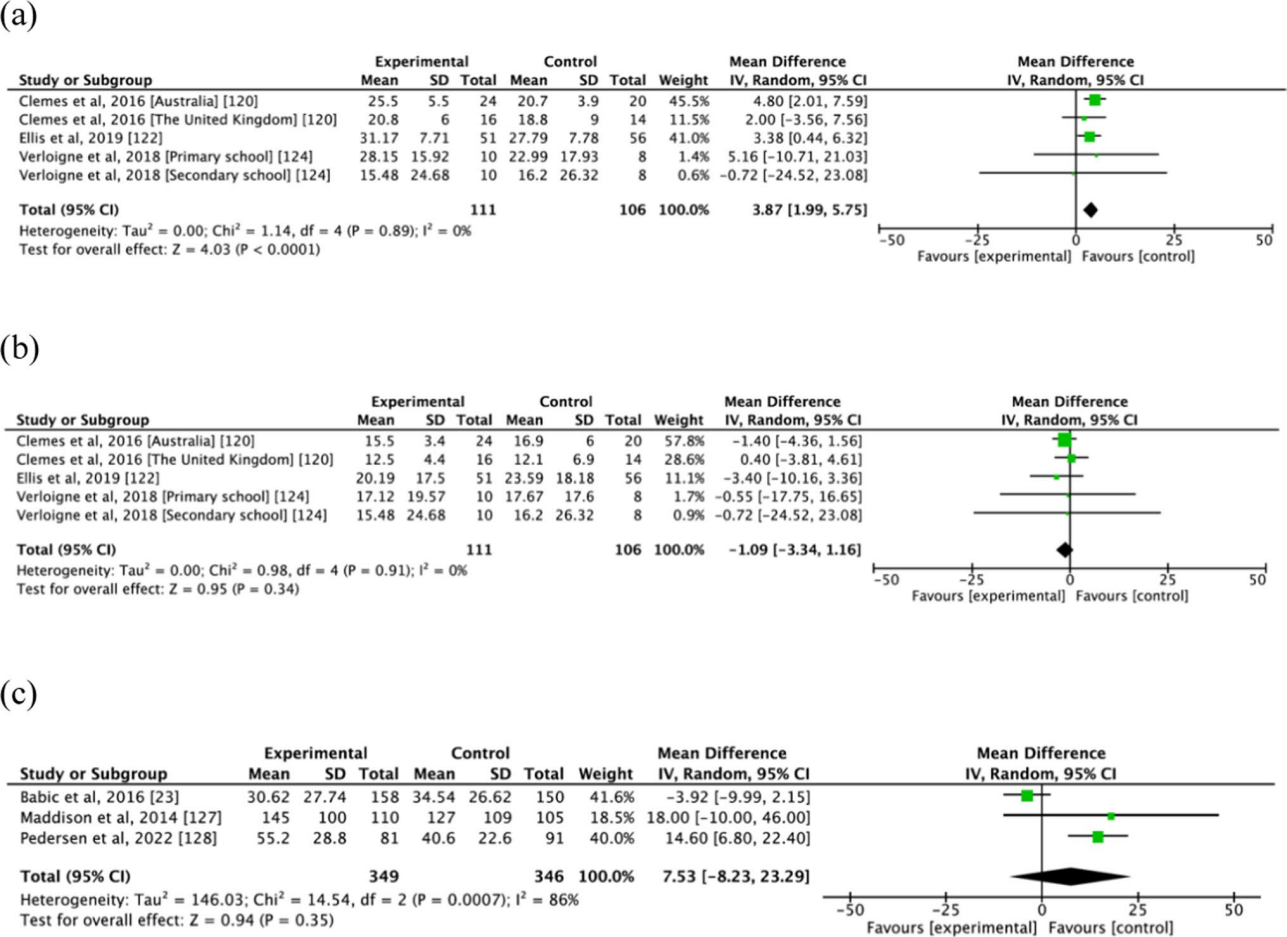
Pooled analysis on the effect of **a** sedentary behaviour intervention on standing (% of wear time), **b** sedentary behaviour intervention on stepping (% of wear time) and **c** screen time intervention on moderate-to-vigorous intensity physical activity (min/day). *CI* confidence interval, *IV* inverse variance, *SD* standard deviation

Of the six studies examining the effect of sedentary behaviour interventions on physical activity [94, 120–124], three reported favourable changes in physical activity in the intervention group [120, 121, 124] relative to the control group; two did not observe any significant differences [122, 123] and one did not examine between-group differences [94]. Based on three included studies that implemented standing desks as a primary intervention strategy and used accelerometers to measure physical activity [120, 122, 124], a meta-analysis of the effect of sedentary behaviour interventions on standing (percent of wear time) reported positive changes (MD = 3.87%, 95% CI 1.99, 5.75, *I*^2^ = 0%) [Fig. 4a]. The same three studies also investigated the effect of sedentary behaviour interventions on stepping [120, 122, 124], but no significant effect was found (Fig. 4b).

### Overflow Effect of Screen Time Interventions on Physical Activity and Sleep

Of the eight studies investigating the effect of screen time interventions on physical activity [22–24, 87, 125–128], six found no difference at post-intervention [22, 23, 87, 125–127], and the remaining two reported increased physical activity in the intervention group compared with the control group [24, 128]. Based on both device-based [23, 128] and subjective measures [127], three studies on the effect of screen time interventions on MVPA (min/day) revealed no significant changes (Fig. 4c). Regarding the effects of screen time interventions on sleep, no significant changes were reported in any of the three studies [22, 127, 128].

### Overflow Effect of Sleep Interventions on Physical Activity and Sedentary Behaviour

Two studies examining the overflow effects of sleep interventions did not find significant changes in physical activity or sedentary behaviour [25, 26]. Both studies were conducted in family-based settings and targeted children below the age of 5 years (i.e. infants, toddlers and preschoolers) [25, 26]. In these studies, parents received educational interventions, and physical activity and sedentary behaviour were measured using accelerometers [25, 26].

### Risk of Bias

Overall, 62 studies were determined to raise some concerns of risk bias, 36 studies were determined to have a high risk of bias, and four studies demonstrated a low risk of bias (Fig. S1 of the ESM) [39, 94, 106, 125]. The main factors contributing to high risk bias were deviations from the intended interventions (e.g. absence of blinding for participants and intervention deliverers, inappropriate analytical methods that failed to address missing data from excluded participants) and measurement of the outcomes (e.g. use of invalid or unreliable measurement tools).

### Sensitivity Analyses and Publication Bias

A series of sensitivity analyses were performed by removing each study one by one from the meta-analysis. The results showed that the pooled effect of physical activity interventions on sedentary behaviour (in minutes per day) in the intervention group became significant, compared with the control group, when either of the two studies was excluded [63, 82]. The findings of the subgroup analyses of the effect of physical activity interventions on sedentary behaviour (percent of wear time) were less robust. Specifically, when one study was removed [45], the previously observed effect was no longer significant in the analysis of the ‘school-aged children’ subsample in the child age subgroup or in the ‘theory-based intervention’ subsample in the theory-based subgroup. Regarding the duration of the interventions, excluding any of the five studies [41, 48, 67, 86, 97] led to non-significant differences between the intervention and control groups in the subsample of interventions that lasted for less than 12 weeks. After removing one study [37], the statistical significance of the overflow effect of physical activity interventions on sedentary behaviour was no longer observed in the ‘ineffective’ interventions subsample. In the sensitivity analyses of the risk of bias findings, removing any of the three studies [41, 48, 61] categorised as ‘high risk of bias’ led to the observed statistical significance of the effect becoming non-significant. Regarding the subgroup analysis examining the effect of physical activity intervention on sedentary behaviour (min/day), based on the measurement, the previously observed effect was no longer significant in the analysis of the ‘subjective measurement’ subsample when one study was removed [95]. The funnel plots did not show any indication of publication bias (Figs. S2 and S3 of the ESM).

## Discussion

This is the first attempt to synthesise evidence on the overflow effects of single movement behaviour interventions on non-targeted behaviours among children aged under 18 years. The findings indicate that physical activity interventions entailed concomitant, albeit small, reductions in sedentary behaviour, as measured by the percentage of wear time using devices, but they had no significant effect on screen time. The impacts may vary across age groups, settings and the effectiveness of the intervention on the target behaviour. Interventions aimed at reducing sedentary behaviour led to increases in standing time but not in stepping time. Interventions designed to reduce screen time did not yield concomitant changes in physical activity or sleep. Findings on the overflow effects of sleep interventions on non-targeted behaviours and of physical activity interventions on sleep outcomes were inconclusive.

Although sedentary behaviour was not targeted in the physical activity interventions in this review, a small decrease in sedentary behaviour was observed, indicating an approximate reduction of 6 min (0.95% of wear time, assuming the accelerometers were worn for 10 h a day). This finding of a decrease in sedentary behaviour is consistent with findings from a previous review [21]. However, that review reported a larger effect size, i.e. a decrease of 32 min in sedentary behaviour, in interventions targeting physical activity in the early years in a sub-group analysis of three studies [21]. This discrepancy in effect size may be attributable to the different inclusion criteria used in the two reviews. Specifically, all three studies included in the latter review compared a specific type of physical activity intervention (i.e. structured lessons, participatory intervention) with control groups that received other physical activity interventions (i.e. free playtime, gym class) [129–131]. However, those studies did not meet the inclusion criteria of this review, specifically that the control group should not receive any intervention.

Our subgroup meta-analyses revealed some trends in studies reporting the effect of physical activity interventions on sedentary behaviour (% of wear time) by age group, setting and the effectiveness of the intervention in changing the target behaviour. The finding that school-aged children and adolescents benefited more from physical activity interventions than preschoolers was consistent with a previous systematic review, which found that older children were more likely to benefit from physical activity in the form of decreasing screen time [132]. The age-related increase in sedentary behaviour among children may explain these differences, as there is more room for improvement among older children [133]. Regarding setting, interventions conducted in schools and childcare centres were more effective in decreasing sedentary behaviour than those conducted in family and community settings. Previous systematic reviews have also observed the favourable effectiveness of school-based interventions on physical activity levels [134], physical activity enjoyment [135] and both physical and mental health [136–138]. Unsurprisingly, although both effective and ineffective physical activity interventions resulted in a decrease in sedentary behaviour, interventions that effectively improved physical activity decreased sedentary behaviour more than interventions that did not effectively improve physical activity. This is plausible because within a fixed 24-h day, an increase in one behaviour (physical activity) is likely to be accompanied by a decrease in other behaviours, such as sedentary behaviour. However, because of the lack of systematic reviews summarising the overflow effects of behaviour change interventions, it was difficult to make comparisons between previous evidence and the present findings.

When sedentary behaviour was expressed as minutes per day, physical activity interventions did not have a significant effect on sedentary behaviour, for both school-aged children and adolescents. However, a subgroup analysis revealed a significant reduction in sedentary behaviour when it was measured subjectively, while the effect was not significant when device-based measurement was used. Importantly, no statistically significant subgroup difference was found between the subjective and device-based measurement approaches. It is noteworthy that a small number of studies (*n* = 4) were included in the ‘subjective measurement’ subsample. Additionally, the risk of bias was rated as ‘some concerns’ or ‘high’ across all the included studies (*n* = 11). Given these limitations, the heterogeneity of measurement should be further explored in future higher quality studies.

With only a limited number of studies (*n* = 7) included in this review and three studies included in the meta-analysis, findings on the effect of physical activity interventions on sleep were inconclusive, with both null findings and favourable changes reported. Unlike sedentary behaviour, the effect of physical activity interventions on sleep may involve various physiological mechanisms in addition to the time allocation due to the fixed total time of a 24-h day. A certain amount of physical activity could modify the functioning of the central nervous system and somatic physiology, and thereby affect the overall mechanisms of sleep [139]. A systematic review and meta-analysis of 66 studies including adult participants observed a small and positive effect size of both acute and regular exercise interventions on sleep duration [140]. The differences between our findings and those of that review may be due to differences in the target population (adults vs children), measurements of sleep outcomes or intervention duration. It is worth noting that all of the interventions included in this review had a duration of less than 24 weeks, and it is plausible that interventions with a longer duration may be necessary to induce changes in sleep patterns. However, there has been no consensus on the minimum duration of interventions required to achieve significant improvements in sleep among both children and adults [141, 142].

Favourable changes in standing time were observed in sedentary behaviour interventions, while no changes were observed in stepping time. It is noteworthy that all three studies included in the meta-analysis of this subgroup implemented standing desks as a primary intervention strategy [120, 122, 124]. A previous systematic review summarising the effect of school-based standing desk interventions among school-aged children and youth aged 5–18 years also reported consistent improvement in standing time [143]. In another systematic review examining the effect of standing desks on step counts among children and adolescents, no significant differences were reported between the intervention and control groups at post-intervention [144]. This could be attributed to the characteristics of standing desks and the context in which they are used (e.g. in a classroom), which primarily substituted standing time for sitting time but may limit opportunities for increasing stepping. To generate an overflow effect on physical activity of other intensities, future sedentary behaviour interventions for children should consider strategies both during and outside classes.

The screen time interventions did not have any significant effect on MVPA. The findings of our meta-analysis were supported by the findings of another intervention involving a large sample (1520 children aged 12–17 years), which did not observe significant changes in the self-reported quantity and frequency of physical activity following an intervention aimed at reducing media use [22]. The strategies used in the various interventions may point to potential reasons for these findings. The included studies aimed to reduce screen time through educational approaches or restrictions on device use [22, 23, 127, 128], rather than by providing alternative activities. As a result, the change in screen time did not necessarily translate into an increase in physical activity, particularly MVPA. However, robust conclusions cannot be made because of the limited number of studies. Regarding the effect of screen time interventions on sleep, no significant effect was observed in this review, but there were a limited number of relevant studies. Our findings were inconsistent with a previous systematic review of 11 studies that reported an approximate 10-min increase in sleep duration among children aged 2–13 years following screen time intervention [145]. The discrepancy between our findings and those of the previous review can be attributed to the different inclusion criteria. Specifically, this review focused exclusively on single-behaviour interventions targeting screen time, whereas the previous review also included multi-behaviour interventions (7/11 studies) and interventions that incorporated strategies to improve sleep (6/11 studies) [145]. Moreover, intervention characteristics are crucial when investigating the effects of regulating children’s screen time on sleep [145]. More specifically, previous interventions have used the strategy of limiting screen use before bedtime to improve sleep [146, 147], whereas all three screen time interventions included in this review focused on reducing total screen time through plans for media use or environmental modifications [22, 127, 128]. Further screen time interventions, especially those focused on evening or pre-bed screen time, are warranted to establish robust conclusions regarding their overflow effect on sleep.

The findings on the overflow effects of sleep interventions, which were based on the narrative analysis of a limited number of studies (*n* = 2), were inconclusive. The overflow effect of sleep interventions for children and adolescents has received limited attention. In a previous systematic review of studies of the effect of sleep interventions on children under the age of 18 years, only one study examined the effect on physical activity, and a sleep component was included as a part of a broader multi-behaviour intervention [148]. Among the two sleep interventions examined in this review [25, 26], one that targeted young children observed an 11-min increase in MVPA within the intervention group, but this was not significantly different from the control group [25]. The second intervention focused on antenatal and early postnatal education, and did not observe significant changes in light-to-vigorous physical activity [26]. This suggests that changes in sleep may not necessarily lead to changes in other types of behaviours. The aims of these sleep interventions should be taken into account, as they usually focus on sleep problems [26] or sleep hygiene [25], rather than sleep duration. Another potential reason for the different findings could be the absence of comprehensive measurement tools for all of the behaviours engaged in during a 24-h day. For instance, one study reported an increase of 54 min in sleep duration and a 2.7-min increase in total physical activity [25]. While not explicitly reported, it is reasonable to anticipate that there was a concomitant decrease in sedentary behaviour among the participants in the same study [25]. In summary, drawing definitive conclusions is challenging because of the limited number of studies.

To the best of our knowledge, this review represents the first attempt to comprehensively summarise the effects of single-movement behaviour interventions on non-targeted behaviours among participants from early childhood to adolescence. Nevertheless, some limitations should be acknowledged. First, in terms of the overflow effects of single-behaviour interventions on sleep and the overflow effects of sleep interventions, a concern arises from the limited number of studies. Second, a discrepancy was found in inclusion criteria for wear time of device-based measurements of the movement behaviours, which may potentially undermine the overall conclusions. Hence, it is critical to reach a consensus on the optimal inclusion criteria for wear time, so that future research can generate more robust evidence. Third, the majority of studies in this review (98/102 studies) were classified as having ‘some concerns’ or a ‘high risk of bias’, which may have contributed to less robust findings. Future high-quality studies with a low risk of bias are needed to strengthen the evidence. Interventions focusing on physical activity among school-aged children and adolescents, and that are implemented in schools and childcare centres appear to yield greater benefits in reducing sedentary behaviour than their counterparts. However, sleep patterns are relatively stable and challenging to modify [149]. More interventions with long durations and objective measurements are needed to examine the effects of behaviour change interventions on sleep. Future interventions are recommended to take holistic 24-h measurements of movement behaviours, regardless of the intervention target, whenever a device-based continuous measurement approach is possible.

Exploring the overflow effects of single-behaviour interventions has multi-faceted clinical and economic implications. Substantial decreases in physical activity and increases in sedentary behaviour are often observed in children and adolescents [150], and movement behaviours such as physical activity and sedentary behaviour have been found to be associated with various health outcomes in this period [151, 152]. While the effect size observed in this review was small, its related clinically important outcomes were supported by previous studies [153, 154]. A study on preschoolers found that replacing 5 min of sedentary behaviour with vigorous-intensity physical activity was linked to higher fat-free mass index, cardiorespiratory fitness and motor fitness [153]. Similarly, a study of school-aged children reported that substituting 10 min of sedentary behaviour with vigorous-intensity physical activity was associated with lower waist circumference and body mass index [154]. Implementing behaviour change interventions during this critical stage may have a positive influence on an individual’s health across the lifespan [155, 156]. The overflow effects of single-behaviour interventions indicate co-dependencies among behaviours, emphasising the need to consider all behaviours holistically. Taking advantage of potential overflow effects may maximise intervention effects. For example, interventions designed to improve physical activity could serve as a strategy to combat both physical inactivity and sedentary behaviour; promoting standing could be a practical alternative for reducing prolonged sedentary time. In the long term, taking advantage of a potential overflow effect can help alleviate economic burdens for both individuals and society. Furthermore, there are emerging efforts to develop interventions that target the three movement behaviours within a 24-h timeframe [157, 158], offering an integrated and flexible approach to achieve similar health outcomes. Nevertheless, it is crucial to acknowledge the complexity of the interplay and trade-offs among these behaviours. It has been argued that different movement behaviours may have unique barriers and facilitators that require tailored intervention strategies [159]. Further studies should help to understand how to optimise health by incorporating multiple target behaviours into an intervention.

## Conclusions

Overflow effects on non-targeted behaviours were observed in interventions aimed at increasing physical activity or reducing sedentary behaviour although the effect size was small. Physical activity interventions effectively reduced sedentary behaviour, with those that improved physical activity showing a greater reduction compared with those that did not. Interventions targeting sedentary behaviour resulted in a concomitant increase in standing time. Overflow effects in other movement behaviours were not observed. These findings shed light on the importance of holistic consideration of a 24-h framework for evaluating movement behaviours. Further multiple-behaviour interventions are needed to explore strategies to obtain optimal health outcomes.

## Supplementary Information

Below is the link to the electronic supplementary material.

Supplementary file1 (PDF 924 KB)

## Declarations

### Availability of Data and Material

All data have been included in the article or included as supplementary information.
