# Supplementary material for: The Overflow Effects of Movement Behaviour Change Interventions for Children and Adolescents: A Systematic Review and Meta-Analysis of Randomised Controlled Trials
Source: Sports Med. 2024 Sep 18;54(12):3151–67. doi: 10.1007/s40279-024-02113-1 (PMC11608165; doi:10.1007/s40279-024-02113-1)
Supplement: Supplementary file 1 — Supplementary file1 (PDF 924 KB) [file 40279_2024_2113_MOESM1_ESM.pdf]

Journal name

Sports Medicine

Article title

The overflow effects of movement behaviour change interventions for children and adolescents: A systematic review and meta-analysis of randomised controlled trials

Author names

Jie Feng, Wendy Yajun Huang, Chen Zheng, Jiao Jiao, Asaduzzaman Khan, Mehwish Nisar, Stephen Heung-Sang Wong

*** Correspondence to**

Wendy Yajun Huang

Department of Sport, Physical Education and Health, Hong Kong Baptist University, Hong Kong, China; wendyhuang@hkbu.edu.hk

Table S1. Search strategy

MEDLINE (Ovid)

**MEDLINE (ovid)**	**13 May 2024**
1. "physical activit*".kf,tw.	137,068
2. exp Sports/	222,907
3. exp Exercise/	256,422
4. ((activ* or outdoor*) adj3 play*).kf,tw.	24,474
5. ("prone position*" or "floor time" or "tummy time").kf,tw.	7,050
6. 1 or 2 or 3 or 4 or 5	457,919
7. Sedentary Behaviour/	14,146
8. (sedentary or inactiv* or (lack adj3 activity)).kf,tw.	360,526
9. ((low adj3 energy expend*) or physical* inactiv*).kf,tw.	11,386
10. sitting.kf,tw.	23,949
11. (chair or stroller or car or automobile or bus or indoor* or in-door).kf,tw.	88,456
12. ("screen time" or screentime or "screen use" or "TV viewing" or television or watch or view or cartoon or "motion picture" or "screen media" or "social media" or "video gam*" or "computer gam*" or "electronic gam*" or smartphone or laptop or ipad or mobile or app or "screen based entertainment").kf,tw.	487,399
13. 7 or 8 or 9 or 10 or 11 or 12	945,115
14. Sleep/	71,239
15. 6 and 13	59,470
16. 13 and 14	4,193
17. 6 and 14	4,908
18. 15 or 16 or 17	65,599
19. ("randomised controlled trial" or RCT or intervention* or "primary prevention" or trial).kf,tw.	1,757,453
20. (newborn or infan* or toddler* or preschool* or pre-school or pediatric or "early year*" or "early childhood" or "young child*" or child* or adolescen* or teen* or young or juvenil* or youth).kf,tw.	2,666,966
21. 18 and 19 and 20	5,222
22. limit 21 to (english language and humans and "all child (0 to 18 years)" and randomised controlled trial)	943

EMBASE (Ovid)

**EMBASE (ovid)**	**13 May 2024**
1. "physical activit*".kf,tw.	220,194
2. exp sport/	221,540
3. exp exercise/	453,151
4. ((activ* or outdoor*) adj3 play*).kf,tw.	36,157
5. ("prone position*" or "floor time" or "tummy time").kf,tw.	13,532
6. 1 or 2 or 3 or 4 or 5	805,431
7. sedentary lifestyle/	22,057
8. (sedentary or inactiv* or (lack adj3 activity)).kf,tw.	475,677
9. ((low adj3 energy expend*) or physical* inactiv*).kf,tw.	18,022
10. sitting.kf,tw.	40,079
11. (chair or stroller or car or automobile or bus or indoor* or in-door).kf,tw.	154,770
12. ("screen time" or screentime or "screen use" or "TV viewing" or television or watch or view or cartoon or "motion picture" or "screen media" or "social media" or "video gam*" or "computer gam*" or "electronic gam*" or smartphone or laptop or ipad or mobile or app or "screen based entertainment").kf,tw.	782,694
13. 7 or 8 or 9 or 10 or 11 or 12	1,431,185
14. sleep/	117,628
15. 6 and 13	98,745
16. 13 and 14	8,285
17. 6 and 14	11,407
18. 15 or 16 or 17	113,071
19. ("randomised controlled trial" or RCT or intervention* or "primary prevention" or trial).kf,tw.	2,970,898
20. (newborn or infan* or toddler* or preschool* or pre-school or pediatric or "early year*" or "early childhood" or "young child*" or child* or adolescen* or teen* or young or juvenil* or youth).kf,tw.	3,768,365
21. 18 and 19 and 20	8,590
22. limit 21 to (human and english language and randomised controlled trial and (infant <to one year> or child <unspecified age> or preschool child <1 to 6 years> or school child <7 to 12 years> or adolescent <13 to 17 years>))	1,173

SPORTDiscus (EBSCO)

**SPORTDiscus (EBSCO)**	**13 May 2024**
S11. S10 with Limiters - Peer Reviewed; Language: English; Publication Type: Academic Journal; Document Type: Article	1,880
S10. S7 AND S8 AND S9	2,268
S9. newborn OR infan* OR toddler* OR preschool* OR pre-school OR pediatric OR "early year*" OR "early childhood" OR "young child*" OR child* OR adolescen* OR teen* OR young OR juvenil* OR youth	270,282
S8. "randomised controlled trial" OR RCT OR intervention* OR "primary prevention" OR trial	146,588
S7. S4 OR S5 OR S6	126,627
S6. S1 AND S3	5,489
S5. S2 AND S3	1,557
S4. S1 AND S2	121,551
S3. sleep	14,773
S2. sedentary OR sitting OR "physical* inactiv*" OR (low n3 energy expend*) OR chair OR stroller OR car OR automobile OR bus OR indoor OR in-door OR sitting OR "screen time" OR screentime OR "screen use" OR "TV viewing" OR television OR watch OR view OR cartoon OR "motion picture" OR "screen media" OR "social media" OR "video gam*" OR "computer gam*" OR "electronic gam*" OR smartphone OR laptop OR ipad OR mobile OR app OR "screen based entertainment"	207,541
S1. "physical activit*" OR sport* OR exercise OR "activ* play" OR "prone position*" OR "floor time" OR "tummy time" OR (activ* or outdoor*) n3 play*	1,223,974

PubMed **13 May 2024**

((((("physical activity"[Text Word] OR "sport*"[Text Word] OR "exercise"[Text Word] OR "active play"[Text Word] OR "prone position*"[Text Word] OR "floor time"[Text Word] OR "tummy time"[Text Word]) AND ("sedentary"[Text Word] OR "sitting"[Text Word] OR "inactive"[Text Word] OR "stroller"[Text Word] OR "screen time"[Text Word] OR "screentime"[Text Word] OR "screen use"[Text Word] OR "TV viewing"[Text Word] OR "television"[Text Word] OR "watch"[Text Word] OR "view"[Text Word] OR "cartoon"[Text Word] OR "motion picture"[Text Word] OR "screen media"[Text Word] OR "social media"[Text Word] OR "video gam*"[Text Word] OR "computer gam*"[Text Word] OR "electronic gam*"[Text Word] OR "smartphone"[Text Word] OR "laptop"[Text Word] OR "ipad"[Text Word] OR "mobile"[Text Word] OR "app"[Text Word] OR "screen based entertainment"[Text Word])) OR (("sedentary"[Text Word] OR "sitting"[Text Word] OR "inactive"[Text Word] OR "stroller"[Text Word] OR "screen time"[Text Word] OR "screentime"[Text Word] OR "screen use"[Text Word] OR "TV viewing"[Text Word] OR "television"[Text Word] OR "watch"[Text Word] OR "view"[Text Word] OR "cartoon"[Text Word] OR "motion picture"[Text Word] OR "screen media"[Text Word] OR "social media"[Text Word] OR "video gam*"[Text Word] OR "computer gam*"[Text Word] OR "electronic gam*"[Text Word] OR "smartphone"[Text Word] OR "laptop"[Text Word] OR "ipad"[Text Word] OR "mobile"[Text Word] OR "app"[Text Word] OR "screen based entertainment"[Text Word]) AND ("sleep"[Text Word]))) OR (("physical activity"[Text Word] OR "sport*"[Text Word] OR "exercise"[Text Word] OR "active play"[Text Word] OR "prone position*"[Text Word] OR "floor time"[Text Word] OR "tummy time"[Text Word]) AND ("sleep"[Text Word]))) AND ("randomised controlled trial"[Text Word] OR "RCT"[Text Word] OR "intervention*"[Text Word] OR "primary prevention"[Text Word] OR "trial"[Text Word])) AND ("newborn"[Text Word] OR "infan*"[Text Word] OR "toddler*"[Text Word] OR "preschool*"[Text Word] OR "pre-school"[Text Word] OR "pediatric"[Text Word] OR "early year*"[Text Word] OR "early childhood"[Text Word] OR "young child*"[Text Word] OR "child*"[Text Word] OR "adolescen*"[Text Word] OR "teen*"[Text Word] OR "young"[Text Word] OR "juvenil*"[Text Word] OR "youth"[Text Word]) Filters: Randomised Controlled Trial, Humans, English, Child: birth-18 years

Results: 1,708

Web of Science

**Web of science**	**13 May 2024**
# 11 (#10) AND LA=(English) AND DT=(Article)	9,093
# 10 #7 AND #8 AND #9	10,868
# 9 AB=(newborn OR infan* OR toddler* OR preschool* OR pre-school OR pediatric OR "early year*" OR "early childhood" OR "young child*" OR child* OR adolescen* OR teen* OR young OR juvenil* OR youth)	3,114,815
# 8 AB=("randomised controlled trial" OR RCT OR intervention* OR "primary prevention" OR trial)	2,589,492
# 7 #4 OR #5 OR #6	207,485
# 6 #1 AND #3	37,850
# 5 #2 AND #3	22,611
# 4 #1 AND #2	161,088
# 3 ALL=(sleep)	403,563
# 2 ALL=(sedentary OR sitting OR inactive OR stroller OR "screen time" OR screentime OR "screen use" OR "TV viewing" OR television OR watch OR view OR cartoon OR "motion picture" OR "screen media" OR "social media" OR "video gam*" OR "computer gam*" OR "electronic gam*" OR smartphone OR laptop OR ipad OR mobile OR app OR "screen based entertainment")	2,965,019
# 1 ALL=("physical activity" OR sport* OR exercise OR "active play" OR "prone position*" OR "floor time" OR "tummy time")	2,147,175

PsycINFO (ProQuest) 13 May 2024

((("physical activity" OR sport* OR exercise OR "active play" OR "prone position*" OR "floor time" OR "tummy time") AND (sedentary OR sitting OR inactive OR stroller OR "screen time" OR screentime OR "screen use" OR "TV viewing" OR television OR watch OR view OR cartoon OR "motion picture" OR "screen media" OR "social media" OR "video gam*" OR "computer gam*" OR "electronic gam*" OR smartphone OR laptop OR ipad OR mobile OR app OR "screen based entertainment")) OR ((sedentary OR sitting OR inactive OR stroller OR "screen time" OR screentime OR "screen use" OR "TV viewing" OR television OR watch OR view OR cartoon OR "motion picture" OR "screen media" OR "social media" OR "video gam*" OR "computer gam*" OR "electronic gam*" OR smartphone OR laptop OR ipad OR mobile OR app OR "screen based entertainment") AND sleep) OR (("physical activity" OR sport* OR exercise OR "active play" OR "prone position*" OR "floor time" OR "tummy time") AND sleep)) AND ("randomised controlled trial" OR RCT OR intervention* OR "primary prevention" OR trial) AND (newborn OR infan* OR toddler* OR preschool* OR pre-school OR pediatric OR "early year*" OR "early childhood" OR "young child*" OR child* OR adolescen* OR teen* OR young OR juvenil* OR youth)

Limited by: Peer reviewed

Record type: Journal Article

Language: English

Age group: Adolescence (13-17 Yrs), Childhood (birth-12 Yrs), Infancy (2-23 Mo), Neonatal (birth-1 Mo), Preschool Age (2-5 Yrs), School Age (6-12 Yrs)

Population: Human

Results: 2,116

Table S2. Characteristics of included studies

Author, year	Country	Study design	Population	Setting	Targeted behaviour	Non-targeted behaviour	Measurement of non-targeted behaviour	Findings (intervention vs. control group)	Intervention duration
Physical activity intervention									
Adamo, 2017	Canada	Cluster RCT, 3-arm	Preschoolers: n = 215, 3.6 yrs, 54% boys	Childcare centre	Physical activity	Sedentary behaviour	Accelerometer	/	6 months
Alhassan, 2022	The United States	Cluster RCT, 2-arm	Toddler: n = 50, 25.0 ± 4.2 months, 58% boys	Childcare centre	Physical activity	Sedentary behaviour	Accelerometer	/	10 weeks
Amini, 2016	Iran	Cluster RCT, 2-arm	School-aged children: n = 334, grades 4-6, 51% boys	School	Physical activity	Screen time	Questionnaire/ question	/	18 weeks
Andersen, 2020	Norway	Cluster RCT, 2-arm	Preschoolers: n = 101, 3.7 ± 0.4 yrs, 43% boys	Childcare centre	Physical activity	Sedentary behaviour	Accelerometer	–	12 weeks
Baquet, 2018	France	Cluster RCT, 2-arm	School-aged children: n = 283, 8.2 yrs, 50% boys	School	Physical activity	Sedentary behaviour	Accelerometer	/	1 year
Beets, 2015	The United States	Cluster RCT, 2-arm	School-aged children: n = 1294, 7.9 yrs, 53% boys	School	Physical activity	Sedentary behaviour	Accelerometer	/	1 year
Blaes, 2013	France	Cluster RCT, 2-arm	School-aged children: n = 332, 8.7 ± 1.6 yrs, 50% boys	School	Physical activity	Sedentary behaviour	Accelerometer	–	Once the playground was changed
Bundy, 2017	Australia	Cluster RCT, 2-arm	School-aged children: n = 221, 6.0 ± 0.6 yrs, 54% boys	School	Physical activity	Sedentary behaviour	Accelerometer	/	13 weeks
Cardon, 2009	Belgium	Cluster RCT, 4-arm	Preschoolers: n = 583, 5.3 ± 0.4 yrs, 52% boys	School	Physical activity	Sedentary behaviour	Accelerometer	/	4-6 weeks
Carlin, 2018	Ireland	Cluster RCT, 2-arm	School-aged children: n = 199, 12.4 ± 0.6 yrs, 0% boys	School	Physical activity	Sedentary behaviour	Accelerometer	/	12 weeks
Comeras-Chueca, 2022	Spain	RCT, 2-arm	School-aged children: n = 29, 10.1 ± 0.8 yrs, 55.2% boys	University, public school	Physical activity	Sedentary behaviour	Accelerometer	?	5 months

Cradock, 2016	The United States	Cluster RCT, 2-arm	Preschoolers and school-aged children: n = 402, 7.7 ± 1.7 yrs, 49% boys	Community	Physical activity	Sedentary behaviour	Accelerometer	/	6 months
Crouter, 2015	The United States	RCT, 2-arm	School-aged children: n = 36, 9.7 ± 0.9 yrs, 53% boys	Community	Physical activity	Sedentary behaviour	Accelerometer	–	10 weeks
Davis, 2006	The United States	Cluster RCT, 3-arm	School-aged children: n = 100, 9.5 ± 1.0 yrs, 41% boys	School	Physical activity	Sleep	Questionnaire/ question	/	13 ± 1.5 weeks
Direito, 2015	New Zealand	Cluster RCT, 3-arm	Adolescents: n = 51, 15.7 ± 1.2 yrs, 43% boys	Family	Physical activity	Sedentary behaviour	Accelerometer	/	8 weeks
Efstathiou, 2016	Greece	Cluster RCT, 3-arm	School-aged children: n = 729, 8-10 yrs, 47% boys	School	Physical activity	Screen time	Questionnaire/ question	/	20 weeks
Engelen, 2013	Australia	Cluster RCT, 2-arm	School-aged children: n = 221, 6.0 ± 0.6 yrs, 54% boys	School	Physical activity	Sedentary behaviour	Accelerometer	–	13 weeks
Essery, 2008	The United States	RCT, 3-arm	Preschoolers: n = 90, 3.1 yrs, 41% boys	Family (online)	Physical activity	Screen time	Questionnaire/ question	/	12 weeks
Evans, 2020	The United States	RCT, 2-arm	School-aged children: n = 94, 9.0 yrs, 49% boys	Camp	Physical activity	Sedentary behaviour	Accelerometer	/	8 weeks
Fantini, 2023	Italy	Cluster RCT, 3-arm	Adolescents: n =144, 14.0 yrs, 35% boys	School	Physical activity	Sleep	Questionnaire/ question	/	4 months
Fetter, 2018	The United States	Cluster RCT, 2-arm	School-aged children: n = 92, 9.5 ± 0.4 yrs, 46% boys	School	Physical activity	Sedentary behaviour	Accelerometer	–	9 months
Gill, 2019	The United States	Cluster RCT, 2-arm	School-aged children: n = 561	School	Physical activity	Sedentary behaviour	Observation (Observing Fitness Instruction Time)	/	2 years
Goldfield, 2016	Canada	Cluster RCT, 2-arm	Preschoolers: n = 83, 3.3 ± 0.6 yrs, 49% boys	Childcare centre	Physical activity	Sedentary behaviour	Accelerometer	–	6 months

Graves, 2010	The United Kingdom	Cluster RCT, 2-arm	School-aged children: n = 58, 9.2 ± 0.5 yrs	Family	Physical activity	Sedentary behaviour	Accelerometer	/	12 weeks
Grendstad, 2024	Norway	RCT, 3-arm	School-aged children: n = 64, 12.3 yrs	Sports club	Physical activity	Sedentary behaviour	Accelerometer	–	8 weeks
Gross, 2017	The United States	Cluster RCT, 2-arm	Infant: n = 456, 3 months, 50% boys	Hospital, health centre	Physical activity	Sedentary behaviour	Questionnaire/ question	/	3 months
Guo, 2015	China	Cluster RCT, 2-arm	School-aged children: n = 41, grades 3-5, 61% boys	School	Physical activity	Sedentary behaviour	Questionnaire/ question	?	1 year
Gutiérrez-Martínez, 2018	The United States	Cluster RCT, 3-arm	Children: n = 120, 10.5 ± 0.6 yrs, 43% boys	School	Physical activity	Sedentary behaviour	Accelerometer	–	10 weeks
Hall, 2022	Australia	Cluster RCT, 2-arm	School-aged children: n = 2485, 8.0 ± 0.8 yrs, 48% boys	School	Physical activity	Sedentary behaviour	Accelerometer	/	1 year
Hands, 2011	Australia	Cluster RCT, 2-arm	School-aged children: n = 303, 87 months, 54% boys	School	Physical activity	Sedentary behaviour	Questionnaire/ question	/	24 weeks
Harrington, 2018	The United Kingdom	Cluster RCT, 2-arm	School-aged children: n = 1752, 12.8 ± 0.8 yrs, 0% boys	School	Physical activity	Sedentary behaviour	Accelerometer	/	14 months
Jauho, 2015	Australia	Cluster RCT, 2-arm	Adolescents: n = 276, 17.9 ± 0.7 yrs, 100% boys	School	Physical activity	Sedentary behaviour	Accelerometer	/	3 months
Johnstone, 2019	The United Kingdom	Cluster RCT, 2-arm	Children: n = 137, 7.1 yrs, 42% boys	School	Physical activity	Sedentary behaviour	Accelerometer	/	10 weeks
Kolle, 2020	Norway	Cluster RCT, 3-arm	Adolescents: n = 1981, 14.0 yrs, 49% boys	School	Physical activity	Sedentary behaviour	Accelerometer	/	3 months
Laukkanen, 2015	Finland	Cluster RCT, 2-arm	Children: n = 91, 6.2 ± 1.1 yrs, 46% boys	Family	Physical activity	Sedentary behaviour	Accelerometer	/	1 year
Leme, 2016	Brazil	Cluster RCT, 2-arm	Adolescents: n = 253, 16.1 ± 0.1 yrs, 0% boys	School	Physical activity	Screen time	Questionnaire/ question	–	6 months

Lerner-Geva, 2015	Israel	Cluster RCT, 3-arm	Preschoolers: n = 204, 4-6 yrs, 51% boys	School	Physical activity	Screen time, sleep	Questionnaire/ question	/	4 months
Li, 2019	China	Cluster RCT, 2-arm	School-aged children: n = 1641, 6.2 yrs, 55% boys	School	Physical activity	Sedentary behaviour	Accelerometer	/	1 year
Likhitweerawong, 2020	Thailand	Cluster RCT, 2-arm	School-aged children and adolescents: n = 70, 13.0 yrs, 69% boys	Hospital, school	Physical activity	Sedentary behaviour, sleep	Questionnaire/ question	/	2 months
Ling, 2024	The United States	Cluster RCT, 2-arm	Preschoolers, n = 95, 4.1 yrs, 42% boys	Childcare centre	Physical activity	Screen time	Questionnaire/ question	–	16 weeks
Madsen, 2015	The United States	Cluster RCT, 2-arm	School-aged children: n = 879, grades 4-5, 49% boys	School	Physical activity	Sedentary behaviour	Accelerometer	/	2 years
Mahajan, 2021	India	Cluster RCT, 2-arm	Adolescents: n = 3707, 13-18 yrs, 49% boys	School	Physical activity	Screen time	Questionnaire/ question	/	2 years
Maloney, 2008	The United States	Cluster RCT, 2-arm	School-aged children: n = 60, 7.5 ± 0.5 yrs, 50% boys	Family	Physical activity	Sedentary behaviour, screen time	Sedentary behaviour: Accelerometer; Screen time: Questionnaire/ question	/	10 weeks
McLellan, 2021	The United Kingdom	Cluster RCT, 2-arm	School-aged children: n = 146, 10.4 ± 0.7 yrs, 45% boys	School	Physical activity	Sedentary behaviour	Accelerometer	–	6 weeks
Mhurchu, 2008	New Zealand	RCT, 2-arm	School-aged children: n = 20, 12 ± 1.5 yrs, 60% boys	Family	Physical activity	Screen time	Log book	–	12 weeks
Moore, 2021	The United States	Cluster RCT, 2-arm	School-aged children: n = 380, grades 2-5	School	Physical activity	Sedentary behaviour	Accelerometer	/	4 months
Morell-Azanza, 2019	Spain	Cluster RCT, 2-arm	Children and adolescents: n = 106, 11.3 ± 2.5 yrs, 38% boys	Family	Physical activity	Sedentary behaviour, sleep	Accelerometer	/	8 weeks
Morgan, 2018	Australia	Cluster RCT, 2-arm	Children and adolescents: n = 153, 7.7 ± 1.8 yrs, 0% boys	Family	Physical activity	Screen time	Questionnaire/ question	–	2 months

Morgan, 2022	Australia	RCT, 2-arm	School-aged children: n = 189, 8.3 ± 1.8 yrs, 0% boys	School + family	Physical activity	Screen time	Questionnaire/ question	–	3 months
Morris, 2019	The United Kingdom	Cluster RCT, 2-arm	Primary children: n = 83, 9.9 yrs, 41% boys	School	Physical activity	Sedentary behaviour	Accelerometer	/	6 weeks
Morrison, 2013	The United Kingdom	RCT, 2-arm	School-aged children: n = 30, 9-11 yrs, 67% boys	Family	Physical activity	Sedentary behaviour	Accelerometer	/	10 weeks
Nathan, 2020	Australia	Cluster RCT, 3-arm	School-aged children: n = 1862, 8.0 yrs, 51% boys	School	Physical activity	Sedentary behaviour	Accelerometer	–	9 months
Nathan, 2021	Australia	Cluster RCT, 2-arm	School-aged children: n = 1847, 8.2 yrs, 52% boys	School	Physical activity	Sedentary behaviour	Accelerometer	/	/
Norris, 2015	The United Kingdom	Cluster RCT, 2-arm	School-aged children: n = 85, 9-10 yrs, 59% boys	School	Physical activity	Sedentary behaviour	Accelerometer	–	2 months
Norris, 2018	The United Kingdom	Cluster RCT, 2-arm	School-aged children: n = 219, 8.6 ± 0.5 yrs, 51% boys	School	Physical activity	Sedentary behaviour	Accelerometer	–	6 weeks
O’Dwyer, 2012	The United Kingdom	Cluster RCT, 2-arm	Preschoolers: n = 76, 3.8 ± 0.6 yrs, 52% boys	Family	Physical activity	Sedentary behaviour	Accelerometer	–	10 weeks
O’Dwyer, 2013	The United Kingdom	Cluster RCT, 3-arm	Preschoolers: n = 240, 4.5 ± 0.6 yrs, 52% boys	School	Physical activity	Sedentary behaviour	Accelerometer	/	6 weeks
Okely, 2020	Australia	Cluster RCT, 2-arm	Preschoolers: n = 658, 3.4 yrs, 52% boys	School	Physical activity	Sedentary behaviour	Accelerometer	/	6 months
Pate, 2016	The United States	Cluster RCT, 2-arm	Preschoolers: n = 379, 4.5 ± 0.4 yrs, 50% boys	School	Physical activity	Sedentary behaviour	Accelerometer	/	6 months
Polo-Recuero, 2023	Spain	RCT, 2-arm	Adolescents: n = 55, 14.9 yrs, 51% boys	School	Physical activity	Sedentary behaviour	Questionnaire/ question	–	10 weeks
Resaland, 2018	Norway	Cluster RCT, 2-arm	School-aged children: n = 1129, 10.2 ± 0.3 yrs, 52% boys	School	Physical activity	Sedentary behaviour	Accelerometer	/	7 months
Riiser, 2020	Norway	Cluster RCT, 2-arm	School-aged children: n = 456, 5-6 yrs, 52% boys	School	Physical activity	Sedentary behaviour	Accelerometer	/	7 months

Riley, 2015	Australia	Cluster RCT, 2-arm	School-aged children: n = 54, 10.5 ± 0.7 yrs, 52% boys	School	Physical activity	Sedentary behaviour	Accelerometer	–	6 weeks
Riley, 2016	Australia	Cluster RCT, 2-arm	School-aged children: n = 240, 11.1 ± 0.7 yrs, 59% boys	School	Physical activity	Sedentary behaviour	Accelerometer	–	6 weeks
Sacher, 2010	The United Kingdom	Cluster RCT, 2-arm	School-aged children: n = 116, 10.3 yrs, 46% boys	Family	Physical activity	Sedentary behaviour	Questionnaire/ question	–	6 months
Saelens, 2002	The United States	Cluster RCT, 2-arm	Adolescents: n = 44, 14.2 ± 1.2 yrs, 59% boys	Family	Physical activity	Sedentary behaviour	Questionnaire/ question	/	4 months
Sahota, 2001	The United Kingdom	Cluster RCT, 2-arm	School-aged children: n = 636, 8.4 yrs, 55% boys	School	Physical activity	Sedentary behaviour	Questionnaire/ question	/	1 year
Sebire, 2018	The United Kingdom	Cluster RCT, 2-arm	Adolescents: n = 740, 12-13 yrs, 0% boys	School	Physical activity	Sedentary behaviour	Accelerometer	–	10 weeks
Simon, 2014	France	Cluster RCT, 2-arm	Adolescents: n = 732, 11.6 yrs, 47% boys	School	Physical activity	Screen time	Questionnaire/ question	–	4 years
Staiano, 2017	The United States	Cluster RCT, 2-arm	Adolescents: n = 37, 15.7 yrs, 0% boys	School	Physical activity	Sedentary behaviour	Accelerometer	/	12 weeks
Strugnell, 2023	Australia	Cluster RCT, 2-arm	School-aged children: n = 1406, 10.7 yrs, 47% boys	Community	Physical activity	Sedentary behaviour	Accelerometer	/	4 years
Suchert, 2015	Germany	Cluster RCT, 2-arm	Adolescents: n = 1162, 13.7 ± 0.7 yrs, 52% boys	School	Physical activity	Sedentary behaviour	Questionnaire/ question	/	12 weeks
Suksong, 2024	Thailand	RCT, 2-arm	School-aged children: n = 42, 10.4 yrs	School	Physical activity	Sedentary behaviour	Accelerometer	/	8 weeks
Sundgot-Borgen, 2019	Norway	Cluster RCT, 2-arm	Adolescents: n = 2446, 16.8 yrs, 43% boys	School	Physical activity	Sleep	Questionnaire/ question	+	3 months
Tarro, 2019	Spain	Cluster RCT, 2-arm	School-aged children: n = 702, 9.2 yrs, 51% boys	School	Physical activity	Screen time	Questionnaire/ question	–	10 months
Taylor, 2018	The United Kingdom	Cluster RCT, 2-arm	Preschool-aged children: n = 239, 9-10 yrs	School	Physical activity	Sedentary behaviour	Accelerometer	–	8 weeks

Toftager, 2014	Denmark	Cluster RCT, 2-arm	Adolescents: n = 1348, 12.5 yrs, 51% boys	School	Physical activity	Sedentary behaviour	Accelerometer	/	2 years
Torres-Lopez, 2024	Spain	RCT, 2-arm	School-aged children: n = 109, 10.1 ± 1.1 yrs, 59% boys	Hospital	Physical activity	Sleep	Accelerometer	/	20 weeks
Trost, 2009	The United States	Cluster RCT, 2-arm	School-aged children: n = 105, 8.0 yrs, 49% boys	Churches	Physical activity	Screen time	Questionnaire/ question	–	4 weeks
Tuominen, 2017	Finland	RCT, 2-arm	Preschoolers: n = 203, 6.5 ± 0.5 yrs, 50% boys	Family	Physical activity	Sedentary behaviour	Accelerometer	/	8 weeks
Vazou, 2018	The United States	Cluster RCT, 2-arm	School-aged children: n = 77, 9.4 ± 0.5 yrs, 53% boys	School	Physical activity	Sedentary behaviour	Accelerometer	/	8 weeks
Verswijveren, 2022	Australia	Cluster RCT, 4-arm	School-aged children: n = 267, 8.7 yrs, 44% boys	School + family	Physical activity	Sedentary behaviour	Accelerometer	?	18 months
Weigensberg, 2021	The United States	Cluster RCT, 4-arm	Adolescents: n = 232, 16.4 ± 0.6 yrs, 34% boys	School	Physical activity	Sedentary behaviour	Accelerometer	/	12 weeks
Weintraub, 2008	The United States	Cluster RCT, 2-arm	School-aged children: n = 21, 10.0 yrs	School	Physical activity	Screen time	Questionnaire/ question	/	6 months
Wells, 2014	The United States	Cluster RCT, 2-arm	School-aged children: n = 227, 9.3 ± 0.7 yrs, 44% boys	School	Physical activity	Sedentary behaviour	Questionnaire/ question	/	1 year
Wolfenden, 2019	Australia	Cluster RCT, 2-arm	Preschoolers: n = 206, 4.1 yrs, 58% boys	Childcare centre	Physical activity	Sedentary behaviour	Accelerometer	/	3 months
Zhou, 2021	China	Cluster RCT, 2-arm	School-aged children: n = 1178, 10.2 ± 0.5 yrs, 57% boys	School	Physical activity	Screen time	Questionnaire/ question	–	1 year
Sedentary behaviour intervention									
Clemes, 2016	The United Kingdom, Australia	Cluster RCT, 2-arm	UK<br>School-aged children: n = 30, 10.0 ± 0.3 yrs, 53% boys<br><br>Australia<br>School-aged children: n =	School	Sedentary behaviour	Physical activity	Accelerometer	+	9 weeks (UK study), 10 weeks (Australian study)

			44, 11.6 ± 0.5 yrs, 43% boys						
Ee, 2018	Australia	Cluster RCT, 2-arm	School-aged children: n = 47,10-11 yrs, 100% males	School	Sedentary behaviour	Physical activity	Accelerometer	+	21 days
Ellis, 2019	Australia	Cluster RCT, 2-arm	Preschoolers: n = 115, 4.1 yrs, 44% boys	Childcare centre	Sedentary behaviour	Physical activity	Accelerometer	/	12 weeks
Parrish, 2018	Australia	Cluster RCT, 2-arm	Adolescents: n = 88, 14.7 ± 0.7 yrs, 50% boys	School	Sedentary behaviour	Physical activity	Accelerometer	/	5 months
Verloigne, 2018	Belgium	Cluster RCT, 2-arm	School-aged children and adolescents: n = 322, 12.9 yrs, 46% boys	School	Sedentary behaviour	Physical activity	Accelerometer	+	6 months
Verswijveren, 2022	Australia	Cluster RCT, 4-arm	School-aged children: n = 267, 8.7 yrs, 44% boys	School + family	Sedentary behaviour	Physical activity	Accelerometer	?	18 months
Screen time intervention									
Babic, 2016	Australia	Cluster RCT, 2-arm	Adolescents: n = 322, 14.4 ± 0.6 yrs, 34% boys	School + family	Screen time	Physical activity	Accelerometer	/	6 months
Epstein, 2008	The United States	Cluster RCT, 2-arm	Preschoolers: n = 70, 5.9 yrs, 53% boys	Family	Screen time	Physical activity	Accelerometer	/	2 years
Lumeng, 2017	The United States	Cluster RCT, 3-arm	Preschoolers: n = 697, 4.1 ± 0.5 yrs, 49% boys	Community	Screen time	Physical activity	Questionnaire/ question	/	4 years
Maddison, 2014	New Zealand	Cluster RCT, 2-arm	School-aged children: n = 251, 11.2 yrs, 57% boys	Family	Screen time	Physical activity, sleep	Questionnaire/ question	/	20 weeks
Moreno, 2021	The United States	Cluster RCT, 2-arm	Adolescents: n = 1520, 14.5 ± 1.6 yrs, 48% boys	Family	Screen time	Physical activity, sleep	Questionnaire/ question	/	3 months
Robinson, 1999	The United States	Cluster RCT, 2-arm	Children: n = 192, 8.9 yrs, 53% boys	Family	Screen time	Physical activity	Questionnaire/ question	/	1 year
Todd, 2008	The United States	RCT, 2-arm	School-aged children: n = 22, 9.9 yrs, 100% boys	Family	Screen time	Physical activity	Log book	+	20 weeks
Pedersen, 2022	Denmark	Cluster RCT, 2-arm	School-aged children: n = 181, 9.1 yrs, 45% boys	Family	Screen time	Physical activity, sleep	Physical activity: Accelerometer	Physical activity + Sleep /	2 weeks

							;<br>Sleep: Single-channel electroencephalography		
Sleep intervention									
Moir, 2016	New Zealand	Cluster RCT, 4-arm	Infants and toddlers: n = 802, 51% boys (women recruited in late pregnancy)	Family	Sleep	Physical activity, sedentary behaviour	Accelerometer	/	2 years
Yoong, 2019	Australia	RCT, 2-arm	Preschoolers: n = 76, 4.4 yrs, 58% boys	Family	Sleep	Physical activity	Accelerometer	/	3 months

Abbreviation: RCT, randomised controlled trial.

+ significantly higher (intervention vs. control group), – significantly lower (intervention vs. control group), / no significance, ? Analysis not conducted to compare the difference between intervention and control groups.

Study	D1	D2	D3	D4	D5	Overall
Adamo, 2017	+	!	+	+	!	!
Alhassan, 2022	+	!	+	+	+	!
Amini, 2016	+	!	+	+	!	!
Andersen, 2020	+	!	+	+	!	!
Babic, 2016	+	!	+	+	+	!
Baquet, 2018	+	-	+	+	+	-
Beets, 2015	!	!	+	+	+	!
Blaes, 2013	!	-	+	+	+	-
Bundy, 2017	+	!	+	+	+	!
Cardon, 2009	!	!	+	+	+	!
Carlin, 2018	+	!	+	+	+	!
Clemes, 2016	+	!	+	+	+	!
Comeras-Chueca, 2022	+	-	+	+	+	-
Cradock, 2016	+	-	+	+	+	-
Crouter, 2015	+	-	+	+	+	-
Davis, 2006	+	!	+	+	+	!
Direito, 2015	+	!	+	+	+	!
Ee, 2018	+	!	+	+	+	!
Efstathiou, 2016	+	!	!	!	+	!
Ellis, 2019	+	!	+	+	+	!
Engelen, 2013	+	-	+	+	+	-
Epstein, 2008	+	+	+	+	+	+
Essery, 2008	+	!	+	!	+	!
Evans, 2020	+	!	+	+	+	!
Fantini, 2023	+	+	+	+	+	+
Fetter, 2018	+	-	+	+	+	-
Gill, 2019	+	!	+	+	+	!
Goldfield, 2016	+	!	+	+	+	!
Graves, 2010	+	!	+	+	+	!
Grendstad, 2024	+	!	+	+	+	!
Gross, 2017	+	!	+	-	+	-
Guo, 2015	+	-	+	+	+	-
Gutiérrez-Martínez, 2018	+	!	+	+	+	!
Hall, 2022	+	!	+	+	+	!
Hands, 2011	+	!	+	+	+	!
Harrington, 2018	+	+	+	+	+	+
Jauho, 2015	+	-	!	+	+	-
Johnstone, 2019	+	-	+	+	+	-
Kolle, 2020	!	-	+	+	+	-
Laukkanen, 2015	!	!	+	+	+	!
Leme, 2016	+	!	+	+	+	!

+ Low risk

! Some concerns

- High risk

D1 Randomisation process

D2 Deviations from the intended interventions

D3 Missing outcome data

D4 Measurement of the outcome

D5 Selection of the reported result

Lerner-Geva, 2015	+	!	+	-	+	-
Li, 2019	+	!	+	+	+	!
Likhitweerawong, 2020	+	!	+	+	+	!
Ling, 2024	+	+	+	+	+	+
Lumeng, 2017	+	!	+	+	+	!
Maddison, 2014	+	!	+	+	+	!
Madsen, 2015	+	!	+	+	+	!
Mahajan, 2021	+	!	+	+	+	!
Maloney, 2008	+	-	+	+	+	-
McLellan, 2021	+	-	+	+	+	-
Moir, 2016	+	!	+	+	+	!
Moore, 2021	+	-	+	+	+	-
Morell-Azanza, 2019	+	-	+	+	+	-
Moreno, 2021	+	!	+	+	+	!
Morgan, 2018	+	!	+	+	+	!
Morgan, 2022	+	!	+	+	+	!
Morris, 2019	+	-	+	+	+	-
Morrison, 2013	+	!	+	+	+	!
Nathan, 2021	+	-	+	+	+	-
Nathan, 2020	+	!	+	+	+	!
Mhurchu, 2008	+	+	+	+	+	+
Norris, 2018	+	-	+	+	+	-
Norris, 2015	+	!	+	+	+	!
O’Dwyer, 2012	+	!	+	+	+	!
O’Dwyer, 2013	+	!	+	+	+	!
Okely, 2020	+	!	+	+	+	!
Parrish, 2018	+	-	+	+	+	-
Pate, 2016	+	!	+	+	+	!
Pedersen, 2022	+	!	+	+	+	!
Polo-Recuero, 2023	+	!	+	+	+	!
Resaland, 2018	+	!	+	+	+	!
Riiser, 2020	+	-	+	+	+	-
Riley, 2016	+	!	+	+	+	!
Riley, 2015	!	!	+	+	+	!
Robinson, 1999	+	!	+	+	+	!
Sacher, 2010	+	-	+	-	+	-
Saelens, 2002	+	!	+	-	+	-
Sahota, 2001	+	!	+	-	+	-
Sebire, 2018	!	!	+	+	+	!
Simon, 2014	+	-	+	-	+	-
Staiano, 2017	+	-	+	+	+	-
Strugnell, 2023	+	!	+	+	+	!

Suchert, 2015	+	!	+	+	+	!
Suksong, 2024	+	!	!	+	+	!
Sundgot-Borgen, 2019	+	-	+	-	+	-
Tarro, 2019	+	!	+	+	+	!
Taylor, 2018	+	-	+	+	+	-
Todd, 2008	+	!	+	-	+	-
Toftager, 2014	+	!	+	+	+	!
Torres-Lopez, 2024	+	!	+	+	+	!
Trost, 2009	+	!	+	+	+	!
Tuominen, 2017	+	!	+	+	+	!
Vazou, 2018	+	!	+	+	+	!
Verloigne, 2018	+	!	+	+	+	!
Verswijveren, 2022	+	+	+	+	+	+
Weigensberg, 2021	+	!	+	+	+	!
Weintraub, 2008	+	!	+	+	+	!
Wells, 2014	+	-	+	+	+	-
Wolfenden, 2019	+	!	+	+	+	!
Yoong, 2019	+	-	+	+	+	-
Zhou, 2021	!	+	+	+	+	!

Figure S1. Risk of bias summary

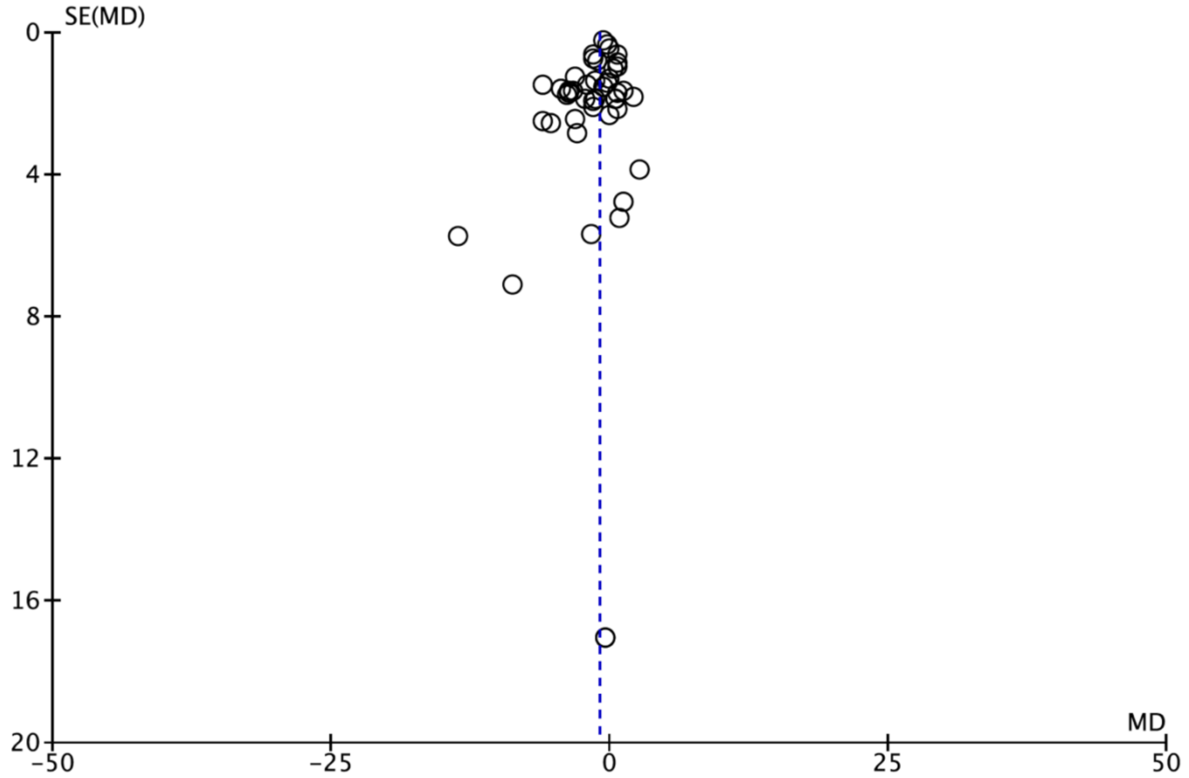

Figure S2. Funnel plot of effect of physical activity intervention on sedentary behaviour (% of wear time)

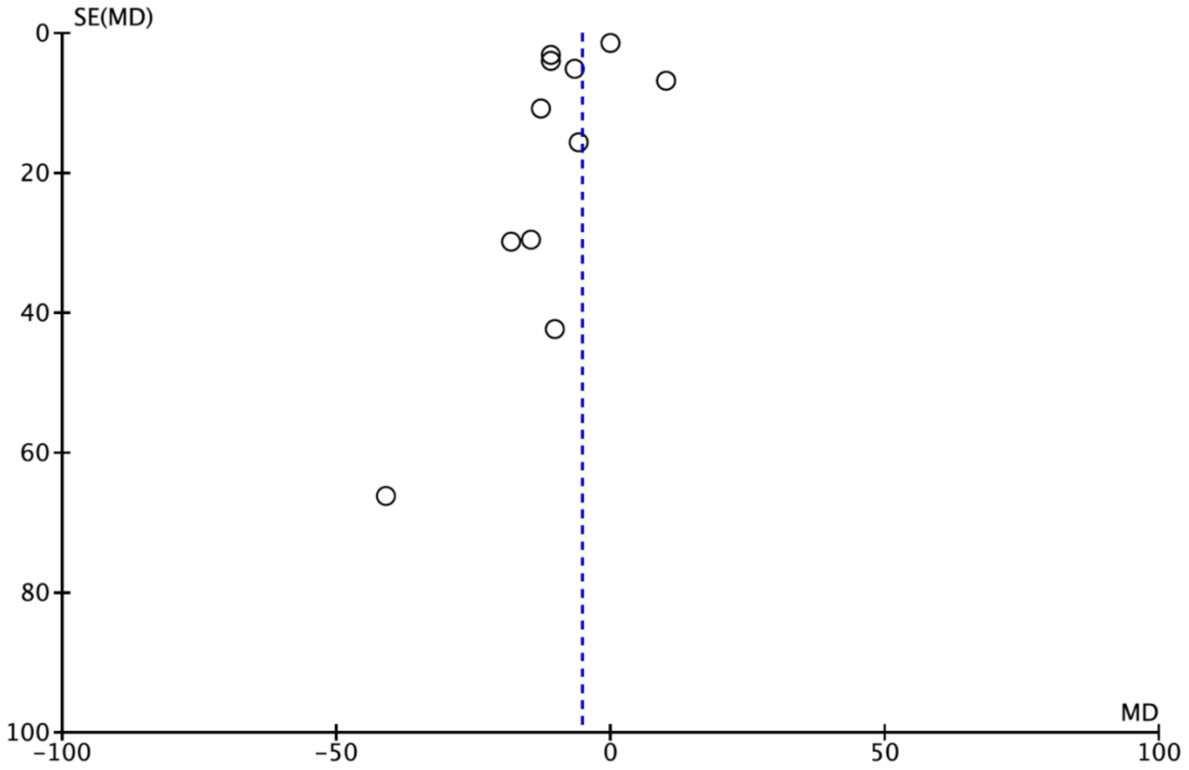

Figure S3. Funnel plot of effect of physical activity intervention on sedentary behaviour (min/day)
